# Parkinson’s disease: current assessment methods and wearable devices for evaluation of movement disorder motor symptoms - a patient and healthcare professional perspective

**DOI:** 10.1186/s12883-020-01996-7

**Published:** 2020-11-18

**Authors:** Ghayth AlMahadin, Ahmad Lotfi, Eva Zysk, Francesco Luke Siena, Marie Mc Carthy, Philip Breedon

**Affiliations:** 1 School of Science and Technology, Nottingham Trent University, Clifton Lane, Nottingham, NG11 8NS UK; 2 Department of Psychology, University of British Columbia in Vancouver, West Mall, Vancouver, V6T 1Z4 Canada; 3 School Of Architecture Design & Built Environment, Nottingham Trent University, Goldsmith Street, Nottingham, NG1 4FQ UK; 4 ICON PLC., South County Business Park, Dublin, Ireland

**Keywords:** Parkinson’s disease, Focus groups, Interviews, Healthcare professionals, Patients, Wearable devices, Qualitative analysis, Preferences

## Abstract

**Background:**

Parkinson’s disease is the second most common long-term chronic, progressive, neurodegenerative disease, affecting more than 10 million people worldwide. There has been a rising interest in wearable devices for evaluation of movement disorder diseases such as Parkinson’s disease due to the limitations in current clinic assessment methods such as Unified Parkinson’s Disease Rating Scale (UPDRS) and the Hoehn and Yahr (HY) scale. However, there are only a few commercial wearable devices available, which, in addition, have had very limited adoption and implementation. This inconsistency may be due to a lack of users’ perspectives in terms of device design and implementation. This study aims to identify the perspectives of healthcare professionals and patients linked to current assessment methods and to identify preferences, and requirements of wearable devices.

**Methods:**

This was a qualitative study using semi-structured interviews followed by focus groups. Transcripts from sessions were analysed using an inductive thematic approach.

**Results:**

It was noted that the well-known assessment process such as Unified Parkinson’s Disease Rating Scale (UPDRS) was not used routinely in clinics since it is time consuming, subjective, inaccurate, infrequent and dependent on patients’ memories. Participants suggested that objective assessment methods are needed to increase the chance of effective treatment. The participants’ perspectives were positive toward using wearable devices, particularly if they were involved in early design stages. Patients emphasized that the devices should be comfortable, but they did not have any concerns regarding device visibility or data privacy transmitted over the internet when it comes to their health. In terms of wearing a monitor, the preferable part of the body for all participants was the wrist. Healthcare professionals stated a need for an economical solution that is easy to interpret. Some design aspects identified by patients included clasps, material choice, and form factor.

**Conclusion:**

The study concluded that current assessment methods are limited. Patients’ and healthcare professionals’ involvement in wearable devices design process has a pivotal role in terms of ultimate user acceptance. This includes the provision of additional functions to the wearable device, such as fall detection and medication reminders, which could be attractive features for patients.

**Supplementary Information:**

The online version contains supplementary material available at (doi:10.1186/s12883-020-01996-7).

## Background

Parkinson’s Disease (PD) is the second most common long-term chronic, progressive, neurodegenerative disease. It mainly affects the motor system, and the cardinal motor symptoms are rest tremor, bradykinesia, rigidity, and postural instability [1–3].

PD prevalence varies from 41 people per 100,000 in the fourth decade of life to over 1900 people per 100,000 in people over 80 years of age [1]. According to the Parkinson’s Foundation [4], more than 10 million people worldwide are living with PD, and about one million people in the United States (US) alone. The annual estimated direct and indirect cost of PD in the US is <DOLLAR/>52 billion [4]. In the United Kingdom, the estimated number in 2018 was around 145,000; that is approximately one adult in every 350 [5].

PD is linked with morbidity, mortality, high economic burden, and a decreased quality of life. However, studies show that positive results can be achieved in the management of motor symptoms in the early stages. The consequences of late or incorrect diagnosis have a negative impact on individuals’ patients and health service system [6–8].

Clinical rating scales are essential to quantify neurological disorders symptoms, impairment and disability [9]. Clinical rating scales in PD enable researchers and clinicians to evaluate disease symptoms, severity, progression, treatment efficiency, response and side effects as well as patients and their caregiver [9–11]. Several clinical rating scales have been developed since the 1960s. The PD rating scales can be categorised into impairment scales, disability scales and multi-modular scales (impairment scales and disability scales) [10]. The most commonly used rating scales of PD motor symptoms are: Hoehn and Yahr (HY) [12] and Unified Parkinson’s Disease Rating Scale (UPDRS) [13].

Hoehn and Yahr (HY) scale was designed originally in 1967 to estimate the disease severity combining deficiency and disability based on bilateral motor involvement and compromised balance and gait [14]. It is a simple scale describes the stage of PD from 1 to 5 based on motor impairment severity and disability. The HY scale has been used widely and has universal acceptance as a scale to describe PD stages due to it is simplicity and ability to group PD patients based on motor and functionality severity and progress [15]. The main advantage of the HY scale is its ease of use, but it is a classification scale and not a rank order [16], i.e., the stage does not reflect the disability as someone in stage 2 may be more impaired for activities of daily living (ADL) than someone in stage 3.

The Unified Parkinson’s Disease Rating Scale (UPDRS) was published in 1987 and become the most widely used rating scale [11]. However, the UPDRS scale has some ambiguities and limitations. The Movement Disorder Society (MDS) has identified some of the limitations, including ambiguities in questions, poor instructions and the absence of important aspects of non-motor symptoms. Their findings have led to revised version MDS-UPDRS to resolve identified problems in the UPDRS and to enable better detection of small changes and mild disabilities [13]. MDS-UPDRS consists of 65 elements requires 30 min administration time distributed among four parts; I) Non-motor experiences of daily living (13 elements), II) Motor experiences of daily living (13 elements), III) Motor examination (33 elements), IV) Motor complications (6 elements) [13]. Each element scored from 0 to 4 where (0: normal, 1: slight, 2: mild, 3: moderate, and 4: severe), some elements are self-administrated by patients without any help, and some elements are completed with or without help from a caregiver, but independently from the examiner, and some elements are rated by the examiner based on observation and physical examination. The MDS-UPDRS scale designed to avoid medical terms to be easier for PD patients, and it applies to PD patients with different levels of disabilities [9].

Even though MDS-UPDRS assessment is internationally accepted rating scale to assess PD, and enhanced the quality of clinical trials outcomes, and has undergone strict clinimetrics validation, but it is clinically based scale, that the clinician assigns numerical scores based on qualitative observations of the patient in various postures and are often insensitive and subjective, so, the assessment depends on the examiners’ skills and knowledge, and it is various from one examiner to another, so examiners’ disagreement on assessment and scores [17–19]. There is evidence showed that MDS-UPDRS has high inter and intra-rater variability between nurses’ and neurologists’ assessments [20–22]. Thus, a patient’s tremor may be assigned MDS-UPDRS score by one examiner and in the next appointment assessed by a different examiner and assigned a higher score. In this situation, it is difficult to interpret these two different scores, whether symptoms worsen or due to subjectivity.

The MDS-UPDRS scale is time-consuming and requires lengthy administration time, approximately 30 minutes, besides it requires specialised official training to improve the coherence of data acquisition and interpretation, these make it unhandy for routine clinical practice [13, 23]. Another time burden that many elements in MDS-UPDRS need to be completed by patients, so additional time is required besides the time required to review these elements by the examiner. This time burden limits the use of the MDS-UPDRS in routine clinical practice. Therefore, MDS-UPDRS scale is mainly used in clinical research. The assessment performed in a clinical environment does not mirror day to day symptoms, which might vary during the day or the last few weeks or months and can only capture a snapshot of patient’s symptoms at that moment [15]. Moreover, many elements of the MDS-UPDRS scale do not apply to each patient, so these questions may raise anxiety in patients, consequently influence their assessment, besides incompetent usage of examiner’s time.

Additionally, many elements in MDS-UPDRS scale depends on patients memory, which is unreliable and limited by recall bias [17–19] due to that fact that most patients are elderly, they exhibit cognition and dementia issues. PD can also cause cognitive dysfunction even in early stages which affect patients recall capabilities [24, 25]. Also, long time between appointments makes it difficult to remember symptoms during this period, besides it is an inconvenience for patients to travel to the clinic due to the weather conditions, transportation, distance, and their conditions, particularly in advanced stages of the disease [26]. Therefore, an early objective and more detailed, accurate, and reliable diagnosis and assessment could be a contributing factor to help increase the chance of effective treatment. This could decrease disability over time, thus cutting down on direct and indirect healthcare costs [3, 27].

Wearable technologies have been increasingly introduced for more accurate motor assessment. They have shown promising results in research and clinical trials to objectively measure and monitor symptoms, both on-site and remotely [18, 28–32]. However, a limited number of commercial systems are available for such purposes [33] such as SENSE-PARK system,^1^ Kinesia system,^2^ Parkinson’s Kinetigraph (PKG)^3^ and Physilog,^4^ and where they do exist, they show limited adoption and implementation [34] despite of the fact that these devices shows high reliability, validity and responsiveness [33, 35], and have been used in the evaluation of PD symptoms and signs by individuals apart from development team and reported successful clinical trials [35, 36]. Data from several studies suggest that these inconsistencies in adoption and implementation may be due to the lack of users’ perspectives in devices design and development [37, 38].

Even though users’ preferences need to be considered if wearable devices are to gain acceptance at home or within a clinic [39, 40], a recent systematic review shows that the research to date has tended to focus on wearable devices development from quantitative perspectives such as type of sensors, data extraction, and classification methods. There is little attention paid to users’ preferences with few studies reporting patients’ or clinicians’ experiences, preferences, and expectations of using wearable devices [31]. here is evidence that remote and continuous monitoring plays a crucial role in treatment quality of patients, as well as reducing costs that afflict the healthcare systems by reducing hospitalizations and increasing patients’ satisfaction.In addition, remote and continuous monitoring makes it possible for physicians to identify disease and its progression early in life and to track changes over time [41], for example, a study conducted by New England Healthcare Institute (NEHI) demonstrated that remote monitoring resulted in a 60% reduction in hospital re-admissions in England with estimated annual saving of up to £6.4 billion [42].

High-quality medical devices with high levels of patients’ and healthcare professionals’ acceptance requires engineering methods such as user-centered design (UCD) philosophy [37, 43] that places an emphasis on patients’ and healthcare professionals’ individual needs as well the environment where the devices will be used. It is suggested that innovations that are often driven by technology evolution may increase the ‘risks’ that researchers develop products that only a few people need and are willing to use [44]. Product development must also take into consideration users’ involvement in the early stages of device design and development where challenges and possibilities, ideas, and concepts are presented and discussed, thus minimising costly device modifications and reducing recalls. This could lead to more robust usable devices that are better suited to users’ needs [38, 44].

A recent systematic literature review was conducted by Johansson et al. [31] found that few studies have explored patients’ preferences or healthcare professionals’ preferences or both [23, 39, 45–56]. However, such studies remain narrow in focus dealing only with device acceptance rather than users’ needs, and in addition, most of these studies have investigated either patients’ perspectives or healthcare professionals’ perspectives and have not included both in the same study. For example, some studies [23, 46, 53] utilized questionnaires to get feedback only from patients linked to existing developed wearable devices and focusing on user satisfaction, comfort and wearing the device publicly. Also, these studies did not involve or explore the design requirements from patients’ point of view or from healthcare professionals. Conversely, some studies explored healthcare professionals’ perspectives, for example, Santiago et al. [52] conducted a survey to evaluate the impact of using a commercially available wearable device (KinetiGraph^™^ or PKG) in routine clinical appointments of PD patients from physicians’ point of view. The study has explored how a developed device could add value to current assessment, but it did not take into consideration the design aspects and what are the requirements since it is limited by feedback about gathered data.

Few studies explored the needs and the requirements of patients and healthcare professionals. For example, Bergmann et al. [39] used an online questionnaire to identify the preferences of medical wearable devices for people affected with arthritis, so these preferences can be used in devices design and developments. However, this study did not take into consideration clinicians’ or physiotherapists’ preferences, which ultimately might limit device adoption. In addition, arthritis conditions such as joint pain might influence arthritis patients’ preferences; for example, the preferable part of the body to place the wearable device, which might be different from other diseases such as PD. Similarly, Bruno et al. [45] used an online survey to identify users’ perspectives toward digital technology and wearable devices, but in this study, people with epilepsy, caregivers, and healthcare professionals were included. However, most of these studies have utilised questionnaires, and approaches of this kind carry with them various well-known limitations [57]. For example, questions understanding and interpretations, the difficulty of conveying feelings and emotions, and they are not flexible and do not allow probing to get in-depth information [58]. In contrast, focus group discussions and interviews can explore a range of views, perceptions, thoughts, and sentiments and can provide insight into complicated subjects [59].

Few studies utilized focused group discussions to gain an in-depth insight into patients’ perspectives, and these can help to design and develop wearable devices with high acceptance and adoption. For example, Papi et al. [50] used focus group discussions to identify design requirements and mode of use of wearable technology for patients with osteoarthritis. Similarly, Thilo et al. [54] and Xing et al. [55] have used the same approach to identify elderly people perspectives toward wearable technologies, but, of these only one study has explored PD patients’ and healthcare professionals’ preferences in-depth, utilizing focus group discussions [49].

Based on an extensive review of the literature and the subsequent completion of the study, we are able to present one of the first comprehensive qualitative studies that has explored both patients’ and healthcare professionals’ perspectives. This is specifically linked to current diagnosis and assessment methods, wearable devices design and materials, and the requirements and specification of a combined PD monitoring solution.

Considering the aforementioned limitations, initially, this study employed semi-structured interviews and focus group discussions to identify the perspectives of healthcare professionals and patients linked to current diagnosis and assessment methods. It also helped to identify their preferences, needs, and requirements of wearable devices to ultimately assess and monitor symptoms of PD. Secondly, their expectations and outlooks on potential solutions.

## Methods

### Data collection

A holistic approach was adopted by first using exploratory semi-structured interviews, and later focus group discussions according to the procedure used by Lambert and Loiselle [60]. The first step in this approach was to conduct four preliminary semi-structured individual interviews for healthcare professionals interviews topic guide, see Additional File 1); three with healthcare professionals (2 females, 1 male; age range: 52-61 years) were recruited from the Royal Derby Hospital, and one interview with 65 years female Parkinson’s local supporter was recruited from Parkinson’s UK, who closely works with PD patients and is understanding of their needs and requirements in different ways such as emotional support, informal discussions about patients worries and experiences. Also, she arranges social events and invites healthcare professional speakers to provide information about PD. This voluntary work enrich her experience and knowledge about PD patients’ requirements. These interviews were utilised to obtain further in-depth information on the current diagnosis and assessment processes. The results (themes) from the exploratory interviews were used to generate a discussion guide for the patient focus groups for patients focus groups topic guide, see Additional File 2). The interview with the local supporter was used to link healthcare professional with patients views and not to generate themes.

The second step was to conduct three focus group discussions involving 12 PD patients (5 females, 7 males; age range: 56-88 years) were recruited through Parkinson’s UK, each lasting approximately 60 minutes. The discussions followed a semi-structured topic guide to allow a deeper insight into product design and development. The topic guide was designed to elicit general discussion with a more specific question as probes used if they were not raised or discussed by participants as per the recommendations of Braun and Clarke [59]. The demographic information of participants is summarised in Table 1.

**Table 1:** Participants demographic information

Data source	Gender Male : Female	Number of participants	Age (year) Mean ± SD (range)	PD Duration (year) Mean ± SD (range)
Focus Group 1	2 : 1	3		
Focus Group 2	3 : 3	6	73.83 ±10.69 (56 - 88)	8.5 ±7.29 (2 - 24)
Focus Group 3	2 : 1	3		
Interviews	1 : 3	4	57.75 ±6.29 (52-65)	NA

### Data analysis

Audio recordings of the healthcare professionals’ interviews and patients’ focus groups were transcribed verbatim by the lead researcher, which enabled initial familiarisation with the data. The data was then analysed using an inductive thematic approach following the six phases guideline as outlined by Braun and Clarke [59]. This involved familiarisation with the data by reading transcripts multiple times and annotating initial ideas and coding interesting elements in the data with a systematic approach by:

1. Organising data in meaningful forms.
2. Grouping codes based on potential themes.
3. Organising data in meaningful forms.
4. Grouping codes based on potential themes.
5. Collecting data relevant to each theme.
6. Reviewing themes by refining themes or sub-themes by splitting, combining themes and find the relationship between themes.
7. Defining and writing up the themes.
8. Final analysis and connect analysis to the research question.

The results from the interviews analysis were utilised to design a focus group topic guide. Verbatim quotes are indicated using the following notation: **I** indicates Interview, **G** indicates focus group, **F** indicates female, **M** indicates Male, and the number indicates the focus group or interview number. For example, GF1 (Female from focus group number one).

## Results and discussions

This study investigated patients’ and healthcare professionals’ opinions toward the current diagnosis and assessment process together with their preferences toward wearable technology and their expectations and outlooks on potential solutions. Limitations of existing solutions and barriers to their use were explored alongside wearable technology design requirements and expected solution outcomes. Both groups of participants showed an unanimously positive response towards the use of wearable technology for remote continuous monitoring. The results from interviews identified the following relevant themes: (1) Current diagnosis and assessment are dubious art, (2) The role of aesthetics and design for acceptance and adoption (3) Patients and healthcare professionals want wearable technology that eases and refines treatments.

### Current diagnosis and assessment methods are dubious art

A common view amongst interviewees was that the current assessment is subjective, dependent on clinical expertise, and thus, inconsistent. Also, there is much scope for Type I (False Positive) and Type II (False Negative) errors in diagnosis.

“***The consultation thing can be a little bit subjective. We doubt each other’s assessment.***” **(IM1)**

“***Sometimes we see patients who have been diagnosed with Parkinson’s disease, but later it turns out to be not Parkinson’s.***” **(IF3)**

”***So, it is about listening to their story, making your own assessment and discussing the options, which might help. It is very much an art.***” **(IF4)**

The interviews show the need for objective data for diagnosis and to distinguish between tremor and other abnormal movements such as essential tremor (ET). Probing questions about the reason behind this suggested that it is very difficult to differentiate PD tremor from ET tremor, especially in early disease stages, which is consistent with other research that has reported a high misdiagnosis rate of PD and ET to be approximately 25% of cases [61].

“**[New assessment method]*Will be able to tell the difference between those types of movements. So, you know, that would be, that will be helpful.***” **(IM1)**

The current assessment and monitoring processes are dependent on patients’ memories and diaries which are not reliable, as previous studies suggested that 30-40% of PD patients will develop dementia [24], besides the fact that most patients are elderly and several lines of evidence suggest that memory decline occurs in individuals older than 60 years [62]. Also, many PD patients are unaware of their symptoms often cannot distinguish between PD signs and other abnormal activities [63].

“**[Patients]*Do not always remember precisely … they*[Neurologists]*might see them six months or even every 12 months …clinically we[Neurologists]asked them to video the movement that they are talking about.***” **(IM1)**

This study supports evidence from previous observations that current diagnosis and assessment scales are subjective, infrequent and depend on clinicians’ skills and patients’ recall [17, 18, 64].

Sometimes consultants ask patients to video tremors they are experience at home for assessment, but elderly patients may often not be familiar with modern technology such as wearable and social computing as exemplified below, and also shown by [65].

“***They do not always know, and a lot of these patients are elderly, and they maybe get the instructions, or they will not know how to video.***” **(IM1)**

A highly surprising fact that emerged from the data was that the health care professionals reported scale or score systems were not commonly used for diagnosis and assessment, including the UPDRS. Probing questions about the reason behind this suggested that assessment scales are time burden and need repetition. This result may be explained by the fact that UPDRS is primarily used for clinical trials and research.

“***I do not give numbers*[scores for the symptoms]*they*** ***[researchers]might do in a research study.***” **(IM1)**

“***We do not tend to use UPDRS routinely in the clinic.***” **(IF4)**

Late diagnosis was a common concern amongst patients, due to late referral from general practitioners (GPs), as most GPs were not suspecting Parkinson’s in the first visits. However, most of the patients were diagnosed correctly and quite quickly when they were examined by a neurologist. Another reported problem was that patients reported having symptoms years before they went to see a doctor or were referred to a specialist.

“***A pain in the shoulder, … the doctor gave me a coat and an injection. That seemed to ease it, but then I needed them. I did not do anything about it for a moment. I just left it, and then I noticed that I was walking, and my right arm was not moving … Then the doctor told me I had a clot on my brain.***” **(GM1)**

“***Dr*[Name]*diagnosed me and provided me a prescription for having some form of, what did he call it? spinal plates.***” **(GM3)**

Concerns were expressed about infrequent assessment; the patients reported that assessments were typically carried out every six months. In addition, assessment depends on a patient’s memory or diary and does not often involve examination or physical assessment. Instead, it mainly focuses on generic subjective patients reports (e.g., “how are you?” “have you got any issues?”). Consistently with healthcare professionals’ reports, none of the participants mentioned UPDRS motor examination. A common view amongst patient interviewees was that infrequent assessment affects their treatment by not taking the right medication and the right dose due to long-time between appointments, and their symptoms might be controlled during the first few months post-assessment but not for the entire six months period. However, all patients reported that they can call nurses between appointments if they feel unwell or if they have any issues.

The patients, on the whole, demonstrated that they experience “peaks and troughs” from hour to hour and from day to day, and some of the symptoms appear at a specific time of the day or when they are doing specific tasks; these patterns are not picked up during clinic assessment.

“***Because I have on and off*[Symptoms fluctuation]*I have started going on and off, so I am m sort of up and down, up and down.***” **(GM1)**

### The role of aesthetics and design for acceptance and adoption

From healthcare professionals’ point of view, wearable devices would be acceptable to most patients, particularly young patients. Device visibility may depend on the stage of the disease; healthcare professionals believed patients in more complex stages of PD would be more likely willing to wear visible devices than would newly diagnosed patients.

“***So, it depends on what stage of Parkinson’s you are talking about. So, if you are going to go to somebody newly diagnosed, they probably want something pretty discreet. But somebody who is in the more maintenance, more complex phase they may wear something that is a bit more visible.***” **(IF3)**

Device design is one of the most important factors that determine whether patients are willing to wear the device. In line with previous studies [23, 39, 48, 49, 54, 56] healthcare professionals felt that the device must be comfortable, easy to use, non-invasive, and should easily be worn under clothes without catching/snagging. The device should also be water-resistant, washable, durable, and easy to fasten to minimise daily disruption.

One main objective of this study was to determine the most preferable and suitable part of the body to wear the device without affecting data quality. All healthcare professionals independently suggested the wrist would be most appropriate, with some focusing on reasons of patient comfort and others on detection of PD tremor characteristics. Given that most typical tremor in PD is called a ‘pill-rolling’ rest tremor involving movement of the thumb and index finger, the wrist would be an appropriate location for the device as it could reliably pick up this type of tremor. These viewpoints match those reported in earlier studies [66, 67].

Healthcare professionals were familiar with what wearable devices are and their use and functionality, and they showed awareness of some commercial devices used for diagnosis and assessment, such as Parkinson’s Kinetigraph (PKG). However, none had ever used these before because they are expensive, and the reports are difficult to interpret. All healthcare professionals reported that wearable devices that available commercially have mostly been used for advanced treatments such as deep brain stimulation and advanced therapies.

“***It is quite expensive. They do use it,*[Neurologist’s name]*in*** ***[City name]he uses them a bit because he is doing, um, he has access to very expensive treatment and so he wants very objective data to give them the expensive treatment … The software that they have developed to interpret the device finding is quite complicated***” **(IM1)**

”***But I think the biggest problem with that is that you have to pay a certain amount of money for every report. Neurologists at*[Hospital name]*use it when they are thinking about advanced therapies***” **(IF4)**

When asked about wearable technology, most patients have not heard about or used wearable technology. Following an explanation of the purposes of this technology as a part of this study, the majority of patients taking part in this study stated that they would be willing to use wearable technology and to be monitored 24/7, as long as the device is not invasive or on an undesired part of body (e.g., neck or ankle). Patients stated the preferred part of the body was the wrist, as one would wear a watch; this was echoed by every participant in this study. This further supports healthcare professionals’ point of view. Also, these results are in keeping with previous observational studies [66, 67].

Issues related to technology such as violation of privacy, difficulty in learning how to use technology, fear and discomfort of using technology and lack of human interaction were not particularly prominent in the discussion. Except for one patient who thought that the fear or dislike of modern technologies (Techno-phobia) could be a barrier for many elderly people, which echoes healthcare professionals’ perspectives. What is interesting about this result is the conflicting perspectives between healthcare professionals and patients. In addition to the growth in the number and proportion of older people [68] suggest that technology acceptance and adoption maybe is not related to techno-phobia, but due to different perspectives and lack of training on new technologies, in addition to technology designs that do not meet users’ needs and requirements.

“***Well, I do not think it is an issue. I mean if it is if somebody is going to try it in the first place to see if it is going to work for everybody … a lot of all the people do not trust modern technology***” **(GM1)**

Regarding wearable design aspects, there are a number of similarities between interviews and focus group discussions in terms of what patients would want, including for the device to be comfortable, non-invasive, waterproof, durable, small, and easy to fasten. Patients’ discussions focused on the wearable hardware side more than did healthcare professionals. For example, patients discussed which materials are more comfortable, breathable, not sweaty/sticky, soft and spongy, including cloth over metal, leather, neoprene, reinforced material, stainless steel, rubber and silicon. Even though they held differing views about what material should be, common ground was to use comfortable material. Moreover, all patients focused on the device wearing style, which be easy to fasten as the tremor affects their ability to fasten traditional clasps such as a buckle or flip styles. Velcro and elasticated straps were the most preferable styles to patients; for example, it was pointed out that Velcro straps could be tightened as needed. Some patients offered it would be better if they were involved in the development stages of the device. These findings are in line with previous studies [23, 39, 48, 49, 54, 56] in which wearable technology’s acceptance was determined by appearance, comfortability, size, and ease of fitting.

“***What about these plastic, leathery straps because this all new stuff coming out. The only thing l would like is a nice easy clasp because that I have got very faster on it, because this is my worst hand so if I have tried to do my watch, that is why I have gone facility like this*[He showed clasp of his watch]*that I can wear it easily, something sticks together***” **(GF3)**

“***I think it might be nice to have an insight into the development stages of whatever the devices that you are going to use so that we can have some input or whether you take it onboard or not***” **(GM1)**

None of the patients had any concerns about device visibility. Perhaps somewhat remarkably, they wanted the device to identify them as people affected by PD, so it might indicate to the community they may need help. Indeed, It has been shown that psychological support would be helpful for people with PD, since emotions affect the severity of the symptoms [69]. Also, it helps those affected avoid unwanted and uncomfortable situations if people know about their disorder, for example, the public might think they are drunk due to the nature of their symptoms. The feeling of embarrassment in public due to PD symptoms has also been described in other studies of PD [70–72].

“***If it is designed to do with Parkinson’s and in time people got to know if they saw that on you, you got Parkinson’s, he might need help***” **(GF3)**

“***That happened to me when I was going through the park. People thought I am the people sitting on a bench though I was drunk they call back to me, told me to take more water with it, you know, just the usual gobby, which in fact that upset me. I never went out actually after that for a few days because it upset me that much***” **(GF2)**

### Patients and healthcare professionals want wearable technology that eases and refines treatments

When asked healthcare professionals about their expectations and outlooks from potentials monitoring and assessment solutions, remarkably all interviewees shed new light on their expectations that the solution could lead for a better or new treatment, and as discussed in “The role of aesthetics and design for acceptance and adoption” section that objective data is needed for expensive treatment as reliable markers; therefore, the solution could help improving current treatments or leading for new ones. The expected solution could be used to evaluate treatments efficacy in terms of medications and rehabilitation.

“***If the nurse altered the medication, and you can detect if the medication reduced patient tremor a bit or knew it did not, or the tremor is bit worse, and that is what you kind of go on, and that is enough, you know, because then you can either try medication try relaxation techniques.***” **(IF3)**

There were some suggestions from healthcare professionals that the solution should be easier to use, provide “***very concise***” information, and be easy to interpret. As mentioned in “The role of aesthetics and design for acceptance and adoption” section, one of the main reasons that interviewed healthcare professionals are not using devices available commercially the complexity of interpreting their data and results.

“***It might be ideal to have something that measured tremor in some way or whether somebody was having an off and on, but it just needs to tell us that quickly and simply, without needing a degree in mastery the charts***” **(IF3)**

”***I think the information it would give would need to be very concise. We would not have time to be going through reams***” **(IF4)**

Concerns regarding lack of information of symptom fluctuations were widespread among healthcare professionals, supporting that symptoms fluctuate from time to time during the day and from day to day. Results show high interest from healthcare professionals in continuous monitoring and its importance in diagnosis and treatment decisions. This finding reflects evidence from previous studies that showed the benefits of continuous monitoring [41, 73].

“***Lots of patients come to us with issues at certain times of the day, whether it is tremor or slowness and stiffness or fatigue or, or you know, being sleepy or you know, all of which could be the symptom of Parkinson’s***” **(IF4)**

“***A pattern to their off time and it might help the nurse how to adjust the medication … If you had something [a solution] that was a bit more technical and a bit more useful in revealing symptoms.***” **(IF3)**

Consistent with the literature [32, 41, 74], this research found that remote monitoring has a pivotal role in PD treatment as well as healthcare cost reduction, mainly for large geographical areas, and it could help patients who are not in the vicinity of a hospital to be diagnosed, particularly in cases of severe symptoms that make travel difficult. Also, it is easier and more efficient use of time for healthcare professionals to assess patients remotely rather than traveling to their homes.

“***Who is living way away, they might not be able to come to the clinic too often.*** [Neurologist’s name]***in*[City name]** ***used it*[Wearable device name]*because I think the geographical area that they cover is quite large***” **(IF3)**

Remote and continuous monitoring was a recurrent topic throughout the discussions; patients seemed to believe it would help clinicians identify their symptom fluctuations and patterns during the day, and if the medication does not help patients to manage their symptoms, continuous monitoring could enable clinicians to change medication in a timely way. Also, it was suggested to track symptom history over a period before a clinic visit, which would ensure that clinicians do not miss any information that might affect patients’ treatment.

“***It would be better if it is 24/7 because then you go to get the full picture of the 24 hours, aren’t you? … Because it is very tiring if you have got Parkinson’s to travel a long way in a day***” **(GF1)**

“***I think if that goes through on that and is something the doctor picks up and appointment comes through to see quite quickly because something in your medication is not working as good as we think it could do.***” **(GF3)**

There were some suggestions to other functionalities to the device, such as fall detection and medication reminders, as this information could help healthcare professionals and patients alike, as forgetting medications and falls are common in PD patients/older age generally [54, 75].

“***Something that counted falls would be useful because patients do not remember how many falls they have … Medication reminder would be useful and does not stop until they have taken it***” **(IF3)**

When asked patients about their expectations and outlooks from potentials monitoring and assessment solutions, a common view amongst patients was that early precise diagnosis and accurate frequent assessment could make their life easier and lead to better treatment, as mentioned earlier by **GF3** and below statement.

“***Something that could make our life easier***” **(GM1)**

“***Privacy sort of goes out the window***” **(GM2)** was the response if the patients have any concern regarding transferring the data over the internet or if someone sees their data or PD information, unlike those in earlier research [45, 49–51, 55]. The current patients seemed to be comfortable forgoing their privacy rights in terms of data access for the overall benefit of treatment. It was also found that patients do not have concerns about device visibility. On the contrary, patients made suggestions the device should be visible to identify them as people affected PD and that they may need help. This finding contrasts with previous studies which emphasize patients’ preferences for the device to be discreet [39, 45, 50].

“***I have Parkinson’s. I am not bothered who knows I got Parkinson’s***” **(GM1)**

“***Privacy, no, I think you lose all your privacy when you have got something like Parkinson’s***” **(GF2)**

Supplementing the health care professionals’ interviews results, it was suggested by patients to add a medication reminder option to the wearable devices and the applications as patients report facing issues with remembering taking the right medication on time. Additionally, patients also suggested a help call button would be useful on the device to summon an emergency contact when needed.

“***I take my medication at set times during the day. Well, this will be able to remind me which medication to take on time***” **(GM2)**

“***I am wondering if you can put something on your watch and, say, I fall in the house or outside. If a press that button it goes straight through to my daughter’s phone and I can speak to her***” **(GF3)**

Previous research has established that user acceptance has a pivotal role in wearable technology adoption [23, 39, 49, 52, 56]; however, very few studies focused on wearable design methods, particularly the utilisation of user-centered design (UCD) philosophy. The current study offers some key insights into user involvement in early design stages and to identify patients’ and healthcare professionals’ requirements and preferences, and the importance of patients and medical professional input was highlighted by participants in this study. Moreover, the prior disregard of such input (alongside the downsides of costliness and difficulty of interpretation of commercially available devices) may help explain the lack of commonplace adoption of wearable technologies. If users are involved in the design process, the device may better suit their needs and overcome any barriers to their use. Some options important to users may have previously been neglected by designers, such as medication reminders and fall detection, which may further interest patients and healthcare professionals to use such wearable devices.

In summary, these results show that current assessment and monitoring processes are subjective and depend on clinicians’ skills and experiences. It was commented that “the consultation thing can be a little subjective”. Also, they are not routinely applied in clinics because they are time-consuming and rely on patients recall, as one interviewee said: “We do not tend to use UPDRS routinely in the clinic”. The participants’ perceptions about using wearable devices to evaluate symptoms were supportive and suggested that this objective evaluation could make the current assessment easier and enhance current treatment. As one participant reported that “he wants very objective data to give them the expensive treatment”. Another important finding was that no concern was raised about wearable devices visibility or private data conveyed through the internet. For example, one interviewee said “Privacy sort of goes out the window”. Participants were interested in participating in device design, and they have proposed many design aspects and options that increase user acceptance and adoption. As one interviewee put it “I think it might be nice to have an insight into the development stages”.

## Study limitations

We acknowledge that this exploratory study has several limitations. First, The sample size may not be fully representative of the wider PD and healthcare provider population. Second, all participants were residing in the Nottingham area. Hence, perceptions may differ in other regions of the world which may limit the generalizability. However, qualitative research rarely seeks to generalize but to explore perceptions. Third, While interviewing participants, we have noticed different levels of knowledge and experience with technologies, so responses were likely based on previous experience with available technologies, such as wearable devices or smartwatches. Therefore, future research should attempt to include participants from different regions, with different experience and knowledge.

## Conclusion

In conclusion, this qualitative study found that current assessment and diagnosis methods are subjective and depends on healthcare professionals’ skills, and this may lead to inconsistent assessment. Currently, there is no general agreement about a reliable, valid, sensitive and cost-effective device to assess PD symptoms. Through this study, healthcare professionals’ and patients’ perspectives of wearable technology were positive and how it could be utilised to improve the current assessment process, thus increase the chance of effective treatment. A common view amongst interviewees was that participation in the design process of wearable devices is a crucial aspect of user acceptance. That involves fitting out additional features for the wearable device, like fall detection and medication alerts, which could be appealing to patients. The outcomes from this research can help to design high-quality wearable devices with a high level of patients’ and healthcare professionals’ acceptance by considering users’ requirements and involving them in the design process.

## Supplementary Information

**Additional file 1** Healthcare Professional Interview Topic Guide. This document contains the semi-structured interviews guide used with healthcare professionals.

**Additional file 2** Patient Focus Group Topic Guide. This document contains the semi-structured focus group guide used with patients.

## Data Availability

The datasets generated and/or analysed during the current study are not publicly available due them containing information that could compromise research participant privacy/consent.

## Abbreviations

UPDRS: Unified Parkinson’s disease rating scale
HY: Hoehn and Yahr
PD: Parkinson’s disease
ADL: Activities of daily living
MDS: Movement disorder society
PKG: Parkinson’s kinetigraph
NEHI: New England healthcare institute
UCD: User-centered design
JICEC: Joint Inter-College ethics committee
ET: Essential tremor
GPs: General practitioners

## Footnotes

1 http://www.sense-park.eu/sense-park-system.php

2 http://glneurotech.com/kinesia/products/kinesia-360/

3 https://globalkineticscorporation.com/the-pkg-system/

4 https://gaitup.com/physilog-sensor/
