# Supplementary material for: Parkinson’s disease: current assessment methods and wearable devices for evaluation of movement disorder motor symptoms - a patient and healthcare professional perspective
Source: BMC Neurol. 2020 Nov 18;20:419. doi: 10.1186/s12883-020-01996-7 (PMC7677815; doi:10.1186/s12883-020-01996-7)
Supplement: Supplementary file 1 — Additional file 1 Healthcare Professional Interview Topic Guide. This document contains the semi-structured interviews guide used with healthcare professionals. [file 12883_2020_1996_MOESM1_ESM.pdf]

Healthcare Professional Interview Topic Guide

Aims:

1- To identify perspectives of healthcare professionals about current methods of diagnosing and monitoring of Parkinson's disease.
2- To identify if healthcare professionals prefer monitored to be done at the clinic or home environment
3- To identify if healthcare professionals prefer wearable or non-wearable devices

Guidance notes:

The participant will be reminded they can terminate their participation at any time, without giving a reason.

Introduction:

1- **The interviewer introduce himself.**
Thank you for giving your time to this research study. My name is ***Ghayth AlMahadin*** *and I am a PhD student in the department of Science and Technology at Nottingham Trent University, researching ways to best measure Parkinson's symptoms.*
2- **Explain the purpose of study.**
   - *In this interview, I will be asking you about your views on current ways to assess and measure tremor in people with Parkinson's disease, and to explore new ways of assessment.*
3- **Remind the interviewee of the participant information sheet he/she has received and ensure that they have read, understood.**
   - *I expect the interview to last about 30-45minutes, is that ok?*
   - *The interview will be recorded and then I will transcribe it to use as data for my research.*
   - *If there are any questions you do not want to answer then that is ok.*
   - *You can stop the interview anytime if you wish*
   - *The questions will be flexible and open-ended to allow you the chance to raise the issues or bring up ideas that you feel are important.*
   - *Your responses will be anonymised in any findings we publish.*
   - *There are no 'right' or 'wrong' answers. We are interested in knowing your opinion.*
   - *Is there anything you would like to ask me before we begin?* **(The interviewer will answer all questions)**.
4- **Ensure that he/she signed the interview consent form.**
5- ***I am now going to start the recording.***

First, I will ask you about the diagnosis and monitoring processes. I would then like to get your opinion about using technology to assist with diagnosis and monitoring of Parkinson's tremor.

Parkinson’s disease diagnosis

1. *Are you trained for PD diagnostic? When did you complete your training?*
2. *How many years of experience do you have in diagnosis of PD?*
3. *How often are you involved in providing the first diagnosis of PD?*
4. *Can you explain the current methods or process you are using for PD diagnosis? How long does the assessment take?*
5. *How many healthcare professionals are typically involved in the diagnosis process at your practice?*
6. *What do you think about these methods? Advantages and disadvantages* ***((probe a few times for disadvantages: “Any others? Any others?”))?*** *What would make it better?*
7. *How accurate are these methods, in your opinion? Why?*
8. *Do you think these methods are subjective or objective? Why?*
9. *In your opinion, what alternatives can be offered for diagnosis? A few years in the future, how would you like to see PD being diagnosed and monitored?*
10. *What is the most noticeable symptom of PD in your opinion?*
11. *What is the most common initial symptom in your opinion?*

Current monitoring approach

1. *Can you describe how PD patients are currently monitored? How often? Where? How long does each monitoring session take?*
2. *How useful do you find current methods? Why?*
3. *In your opinion, do you think these sessions are enough? Should there be more or fewer?*
4. *What is your opinion on the importance of monitoring PD symptoms? Is it related to patients’ treatment, and if so, how?*
5. *What do you think about the current monitoring process? How could it be made it better?*
6. *Can you think of any alternatives that could be developed in the future to assess and/or monitor PD?*
7. *If there was technology that could easily be used for assisting monitoring and/or diagnosis, what would be your opinion on health care professionals’ adoption of this?*

Wearable technology

The interviewer will explains wearable technology and answer any questions.

1. *How would you feel about monitoring patients’ conditions at home using a wearable device? Would you be interested in this kind of technology? Would you use it? Why or why not?*
2. *If you could monitor patients’ tremors using a wearable device, how do you think the device should look and feel? why?*
3. *Are there any alternative options to these wearable devices in your opinion? What are they?*
4. *What part of body would be best to wear the device for tremor measurement, and why?*
5. *Do you have any concerns about such device? Explain please.*
6. *Do you think it would better to use the technology for monitoring or diagnosis? Why?*
7. *What is your opinion on the device collecting data all day and night 24/7? If not why? If yes, what do you think about sending data to the clinic over internet?* **If they have any concerns (e.g. Security of data), probe for that..**
8. *What type of data or information could help you to assess the tremors?*
9. *Would this kind of technology interest you? Would you use it? Why?*

10. *What improvements could be made to the device that could make it more useful?*
11. *Do you think the patients will engage with this technology and wear the device? Why or why not? (you may need to explain the level of engagement that would be needed by the patient)*

Closing

1. *Is there anything else you would like to say about what we have discussed? Do you have any questions?*

Thank you for your time and useful participation
