# Supplementary material for: Parkinson’s disease: current assessment methods and wearable devices for evaluation of movement disorder motor symptoms - a patient and healthcare professional perspective
Source: BMC Neurol. 2020 Nov 18;20:419. doi: 10.1186/s12883-020-01996-7 (PMC7677815; doi:10.1186/s12883-020-01996-7)
Supplement: Supplementary file 2 — Additional file 2 Patient Focus Group Topic Guide. This document contains the semi-structured focus group guide used with patients. [file 12883_2020_1996_MOESM2_ESM.pdf]

Patients Focus Group Topic Guide

Aims:

1- To identify perspectives of patients with current methods to diagnosis and monitor of Parkinson's disease.
2- To identify if patients prefer to be monitored within clinic or at home.
3- To identify if patients prefer wearable or non-wearable devices

Guidance notes:

The participant will be reminded they can terminate their participation at any time, without giving a reason.

Introduction:

1- **The researcher introduce himself.**
Thank you for giving your time to this research study. My name is Ghayth AlMahadin and I am a PhD student in the department of Science and Technology at Nottingham Trent University, researching ways to best measure Parkinson's symptoms.
2- **Explain the purpose of study.**
- *In this group we will discuss your views on current ways to assess and measure tremor in people with Parkinson's disease, and to explore new ways of assessment.*

3- **Ensure that all participants have received, read and understood the participant information sheet.**
- *I expect the discussion to last about 100 minutes,* and ***you can leave at any time with no reason given***. *is that ok?*
- *The discussions will be recorded and then I will transcribe it to use as data for my research.*
- *You can withdraw from the discussion anytime if you wish*
- *The questions will be flexible and open-ended to allow you the chance to raise the issues or bring up ideas that you feel are important.*
- *Your responses will be anonymised in any findings we publish.*
- *There are no 'right' or 'wrong' answers. We are interested in knowing your opinion.*
- *Is there anything you would like to ask me before we begin?*

4- **Ensure that all participants signed the consent form.**
5- ***I am now going to start the recording***

First, I will ask you about the diagnosis and monitoring processes. I would then like to get your opinion about using technology to assist with diagnosis and monitoring of Parkinson's tremor.

Parkinson's disease diagnosis

1. *What was the first symptom of PD you noticed?*
2. *Can you describe the diagnosis process and your experience of it?*
3. *What do you think about diagnosis process? What would make it better?*
4. *In your opinion, what alternatives could or should be offered for diagnosis?*

Current monitoring approach

1. *Can you describe how is your condition is currently monitored? How often? Where?*
2. *Would you like to attend more or less often than you do now? Why? Are there any issue you face when you attend these sessions?*
3. *What is your opinion on the importance of monitoring PD symptoms? Is it related to your treatment, and if so, how?*
4. *What do you think about the current monitoring process? How could it be made better?*
5. *What alternatives would you like to see in the future? why might these be useful?*
6. *If there was technology that could easily be used for assisting monitoring and/or diagnosis, what would be your opinion on health care professionals' adoption of this?*

Wearable technology

The researcher will explain wearable technology and answer any questions.

1. *How would you feel about monitoring your conditions at home using a wearable device? would you be interested in this kind of technology? Would you use it? Why/why not?*
2. *If the doctors can monitor your condition using a wearable device, how do you think the device should look and feel? why?*
3. *What part of body will you prefer to wear the device (wrist, hand, arm) and why?*
4. *Are there any alternative options to wearable devices in your opinion? What are they?*
5. *Do you have any concerns about device visibility?*
6. *How long do you think you would be willing to wear such a device, and why? What if the device looks and feels like a watch?*
7. *What is your opinion on the device collecting data all day and night 24/7? If not, why? If yes, what do you think about sending data to the clinic over internet?* **If they have any concerns (e.g. Security of data).**

Closing

1. *Is there anything else you would like to say about what we have discussed? Do you have any questions?*

Thank you for your time and useful participation
